# Network analysis of clinical features in patients with treatment-resistant schizophrenia

**DOI:** 10.3389/fpsyt.2025.1537418

**Published:** 2025-02-06

**Authors:** Wei Li, Jing Zhao, Na Hu, Wanling Zhang

**Affiliations:** ^1^ Beijing Huilongguan Hospital, Peking University Huilongguan Clinical Medical School, Beijing, China; ^2^ College of Art and Design, Beijing University of Technology, Beijing, China; ^3^ Department of Psychosomatic Medicine, Beijing Children’s Hospital, Capital Medical University, National Center for Children Healthy, Beijing, China

**Keywords:** TRS, NTRS, network analysis, psychopathological symptoms, clinical features

## Abstract

**Objective:**

This study compares the clinical features of Treatment-Resistant Schizophrenia (TRS) and Non-Treatment-Resistant Schizophrenia (NTRS) using network analysis.

**Methods:**

We recruited 511 patients, dividing them into TRS (N = 269) and NTRS (N = 242) groups. Eight scales were used: Positive and Negative Syndrome Scale (PANSS), Positive Symptom Assessment Scale (SAPS), Scale for Assessment of Negative Symptoms (SANS), Simpson-Angus Scale (SAS), Abnormal Involuntary Movements Scale (AIMS), Barnes Akathisia Rating Scale (BARS), Calgary Schizophrenia Depression Scale (CDSS), and Global Assessment of Functioning Scale (GAF). Demographic and clinical data were analyzed using T-tests and Chi-square tests. Network analysis was then applied to compare clinical features.

**Results:**

Significant differences were found in the overall architectures (S = 1.396, *p* < 0.002) and edge weights (M = 0.289, *p* < 0.009) of TRS and NTRS networks. Nine edges (*p* < 0.05) and five nodes (p < 0.01) differed, indicating a correlation between clinical symptoms of the two groups. TRS core symptoms were linked to social functions through both positive (SAPS) and negative symptoms (SANS), while NTRS core symptoms were related to general psychopathological symptoms (PANSS-G).

**Conclusion:**

For TRS, it is essential to address both negative and positive symptoms, focusing on the impact of negative symptoms on functioning. Additionally, managing medication side effects is crucial to avoid worsening negative symptoms.

## 1 Introduction

Treatment-resistant schizophrenia (TRS) is generally defined as failure to respond to treatment with two antipsychotic drugs in sufficient doses and duration, without respond adequately to antipsychotic drugs (1, 2). TRS is characterized by persistent positive symptoms (hallucinations and delusions), negative symptoms (social withdrawal and apathy), and cognitive impairments despite treatment efforts (3). It is estimated that the prevalence of TRS is about 20-30% of those diagnosed with schizophrenia (4). This subset of patients typically experiences more severe symptoms, a longer course of disease, and a higher incidence of comorbidities than patients who respond to treatment (5).

Meanwhile, TRS severely affects their social networks and family members, which can lead to social issues (unemployment and homelessness) and increase medical burden (6). Currently, there are no proven effective treatment strategies for TRS, and investigation of the clinical characteristics of TRS may help in the selection of interventions. There is, therefore a paramount pressing clinic need to distinguish between treatment and non- treatment resistance schizophrenia, providing tailoring treatments to reduce and eliminate drug resistance and improve patient prognosis, further to understand pathophysiological mechanisms of schizophrenia, contributing to the broader field of psychiatric research (7, 8).

Despite significant advances in clinical diagnostic, the heterogeneity can lead to discrepancies in prevalence rates and complicates the identification of true TRS cases. Network analysis has been increasingly applied in psychological illness studies and schizophrenia (9, 10), to explore the core symptom and investigate the relationships between symptoms (11). Cross-sectional network analyses have supported a complex structure of multiple domains for negative, positive and psychological symptoms (11, 12). Psychological networks are composed of nodes that symbolize observed symptoms, linked by edges that illustrate statistical relationships. This method aids in identifying crucial information or key symptoms pertinent to clinical status or patient prognosis, providing an alternative to depending solely on overall scores from scales or categorical diagnoses (13).

Although previous studies have found the symptom networks of schizophrenia, and investigate predictors of TRS and found that a number of clinical features (early age of onset, poor pre-illness social functioning, and a longer period of untreated psychosis) are associated with TRS (14–16). There are also inconsistencies in the findings, such as whether male sex predicts refractory schizophrenia, with some studies showing different conclusions (16, 17). Negative symptoms are often associated with limited drug response and significantly deteriorate function and quality of life in patients with schizophrenia (7). In our previous studies, we have found a strong relationship between negative symptoms and some secondary factors, especially positive symptoms and depressive symptoms (18). It can be seen that negative symptoms may be related to TRS. However, the mechanism of the symptomatic interaction remains unclear. Given the complex relationship between symptoms in schizophrenia, further research is needed to explore the relationship between symptoms investigated core symptom of TRS and distinguished the differences between TRS and NTRS (19). The application of network analysis in TRS symptom analysis may deepen our understanding of TRS.

The main purpose of this study is twofold. First, our goal was to investigate the differences between TRS and NTRS in terms of clinical features and contributing factors. Second, through network analysis, we aimed to present the correlation between clinical features in the two groups to further explore the relationship and mutual influence between TRS clinical features. We hypothesized that TRS would exhibit more severe and complex interrelationships between clinical features.

## 2 Methods

### 2.1 Participants

A cross-sectional design was used to conduct a sample survey at Huilongguan Hospital in Beijing from May 1, 2023 to May 1, 2024. According to the fifth edition of the Diagnostic and Statistical Manual of Mental Disorders (DSM-V), schizophrenia patients were recruited from the inpatients of Beijing Huilongguan Hospital. A total of 511 patients were included in the study, including 269 patients with TRS and 242 patients with NTRS. Patients with TRS were defined as (4): little responding to two or more different antipsychotic medications ≥ 600mg/d chlorpromazine (CPZ) -equivalent dose for at least 6 weeks; Summary Psychiatric Rating Scale (BPRS) total score ≥45 points; Clinical Global Impression - Disease Severity (CGI-SI) scale score ≥4 points. Patients with NTRS were defined as having a good clinical response to antipsychotic medications with a CGI-SI score of < 3 for at least 12 weeks.

### 2.2 Inclusion and exclusion criteria

The inclusion criteria of this study were: (1) Diagnosis of Schizophrenia based on the criteria in the DSM-5; (2) Age between 18 and 60 years; (3) Stable antipsychotic treatment for at least 6 weeks, without any changes in medication during this period; (4) voluntary participation and signing of informed consent. The exclusion criteria were: (1) Comorbidity with other major mental disorders (e.g., bipolar disorder, major depressive disorder); (2) Presence of significant neurological conditions (e.g., brain tumors, stroke) or serious and unstable physical diseases that could interfere with the study; (3) Pregnancy or breastfeeding; (4) Current substance abuse (including drugs or alcohol); (5) Received modified electroconvulsive therapy (ECT) within the past 3 months; (6) Inability to complete all study assessments or cooperate with the study protocol (e.g., due to cognitive impairment). Obtain informed consent from all patients and their guardians. This study was approved by the Ethics Committee of Beijing Huilongguan Hospital (2023-46).

### 2.3 Assessment tools

#### 2.3.1 The positive and negative syndrome scale

PANSS is a widely used tool to assess the severity of schizophrenia symptoms (20). The Chinese version of PANSS consists of 30 items divided into three subscales: positive, negative, and general psychopathology. The severity of each item was rated on a Likert scale from 1(none) to 7(extreme), with higher scores indicating more severe symptoms. The Chinese version of PANSS has been shown to be a reliable and effective assessment tool for assessing psychopathological severity in hospitalized, stable patients with schizophrenia (21).

#### 2.3.2 The scale for assessment of negative symptoms

SANS is a well-established tool for assessing negative symptoms in five domains, including affective flattening, alogia, avolition apathy, anhedonia-asociality, and attention. The scale consisted of 25 items, rated on a 6-point Likert scale ranging from 0(none) to 5(extreme), with higher scores indicating more negative symptoms and greater impairment (22). The Chinese version of SANS has been proven to have good reliability and validity (23).

The most recent view of negative symptoms in patients with schizophrenia indicates that some items in SANS do not fall under the category of negative symptoms, including attention deficit (SANS overall rating of attention), inappropriate emotion (SANS item 6), and poor verbal content (SANS item 10) (24, 25). We used the SANS excluding the above items for the data analysis in this article.

#### 2.3.3 The assessment of positive symptoms

SAPS consists of 34 items that assess positive symptoms in four areas of schizophrenia, including hallucinations, delusions, bizarre behavior, and positive formal thought disorder (22). Each item is rated on a severity scale from 0(non-existent) to 5(extremely serious). In addition, each subscale has its own overall score, ranging from 0(non-existent) to 5(extremely severe), assessing the overall severity of each symptom area. The total SAPS score is the sum of all questions except the global questions, and the total SAPS score is the sum of the four global questions. The Chinese version of SAPS has good reliability and validity (23).

#### 2.3.4 The Simpson–Angus scale

SAS is a proven and sensitive tool for evaluating extrapyramidal side effects caused by antipsychotics (26). The scale consists of 10 items, rated on a 5-point Likert scale, with each item scored from 0 to 4. A higher SAS score indicates more severe extrapyramidal side effects.

#### 2.3.5 Abnormal involuntary movements scale

AIMS is a well-established and validated scale for the evaluation of abnormal involuntary movements, with a particular focus on tardive dyskinesia (TD) (27). The AIMS consists of 12 items, graded from 0 to 4, that tap into abnormal involuntary movements, mainly TD. The scale comprises of 4 questions about oral-facial movements, 3 questions about limb and trunk movements, 3 questions about overall judgment, and 2 questions about dental condition. AIMS is a validated measure for evaluating TD and its treatment effects (28).

#### 2.3.6 The Barnes akathisia rating scale

BARS is a validated tool used to rate drug-induced akathisia, which includes diagnostic criteria for both pseudoakathisia and mild, moderate, and severe akathisia (29). The scale consists of items for assessing observable restless movements, subjective awareness of restlessness, and any associated distress. Each item is rated on a four-point scale from 0 to 3, with a total score range of 0-9. Additionally, a global severity item is included and rated on a six-point scale from 0 to 5. The BARS has good face validity and reliability and is a useful tool for assessing the severity of akathisia (30).

#### 2.3.7 Calgary depression scale for schizophrenia

CDSS was developed to assess depressive symptoms in patients with schizophrenia. It consisted of nine items with a Likert score of 0-3 for each item, with higher scores indicating more severe depressive symptoms (31). The Chinese version of CDSS is a valid and reliable instrument for the assessment of depression in schizophrenia (32).

#### 2.3.8 Global assessment of functioning scale

The GAF is a widely used scale for assessing the overall functioning of patients with schizophrenia, including their psychological, social, and occupational well-being (33). The scale ranges from 1 to 100, with higher scores indicating better functional retention and milder symptoms. The GAF is subdivided into ten 10-point intervals, and its use is common in both research and clinical practice. Studies have demonstrated the reliability and validity of the GAF as a valuable objective indicator of overall patient functioning (34).

In addition, BPRS (Brief Psychiatric Rating Scale) is used tool to assess the severity of psychiatric symptoms (35). The Chinese version of BPRS consists of 18 items that measure various dimensions of psychiatric symptoms, including thought disturbance, emotional withdrawal, and hostility (23). Each item is rated on a Likert scale from 1 (not present) to 7 (extremely severe), with higher scores indicating more severe symptoms. The Chinese version of BPRS has been demonstrated to be a reliable and effective assessment tool for evaluating psychiatric severity in hospitalized patients with various mental health disorders. The CGI (Clinical Global Impression) scale is a widely used tool to assess the overall severity and improvement of psychiatric symptoms (36). The CGI consists of three subscales: Severity of Illness (CGI-SI), Global Improvement (CGI-I), and Efficacy Index. Each item is rated on a scale from 1 (normal, not at all ill) to 7 (among the most extremely ill patients), with higher scores indicating more severe symptoms or greater improvement. The CGI scale has been demonstrated to be a reliable and effective assessment tool for evaluating the overall clinical impression of patients’ psychiatric conditions. The scores of BPRS and CGI-ISI were used to identified the patients with TRS.

Four professional psychiatrists with training and consistency assessment (intra-class correlation coefficient of 0.90) conducted on-site assessments of the schizophrenia scale and collected survey results. Each eligible patient was evaluated by two psychiatrists on the same day. The first psychiatrist assessed the patients and collected their socio-demographic information, clinical data, and psychiatric history through questionnaires. A second psychiatrist tested the patient on all eight scales.

### 2.4 Data analysis

#### 2.4.1 Descriptive analysis

To ascertain the differences in demographic and clinical data between TRS and NTRS, independent-sample t-tests and Chi-square tests in SPSS (Version 26.0) was performed. Quantitative data were described using Mean (M) and Standard Deviation (SD) or N, and with a two-tailed significance threshold of 0.05.

#### 2.4.2 Network estimation

Based on the inclusion and exclusion criteria, the subgroups of total sample (N= 511) were diagnosed with TRS (N = 269) and NTRS (N = 242). We utilized the R-package qgraph in R (version 4.2.2) (37) to load to construct the networks and determine the centrality indexes. This program applied a Gaussian graphical model (GGM) to examine data of two networks (TRS and NTRS) derived from a dataset of 511 participants. The regularized causal association network (shrinkage and selection operations) process is constructed by using GGM model algorithm, which was set at the advised value of 0.5 (38) based on minimum absolute glass graph (LASSO) (39). According to LASSO regularization, all the edges are shrunk precisely to zero to reduce the pseudo-correlation (40), and the optimal fitting model was selected using Extended Bayesian Information criteria (EBIC) (41). Within the illustrated network, red and blue edges depicted negative and positive partial correlations.The thickness of an edge represented the strength of the correlational coefficient. We set a minimum weight threshold of 0.1 for each edge to provide a clear visualization of the network.

Using a centrality metric to describe the connectivity of each node can determine which symptoms are more important, or more impactful in the network. 10 Nodes respectively representing scores of each scale were calculated to construct TRS and NTRS networks separately. Then the network comparison test (NCT) was employed, contrasting the established TRS and NTRS networks to determine whether differences existed between their global and edge strengths.

The “bootnet” function in R was employed to gauge the network’s stability and accuracy (42). The accuracy of edge weights was evaluated using 95% confidence intervals (CI) calculated by bootstrap techniques. A slimmer CI indicates a more accurate estimate of the edge weights and centrality indicators. The stability of the centrality measures was evaluated by figuring out the correlation stability coefficient (CS-coefficient) through a case-dropping bootstrap technique. Ideally, CS-coefficient, indicating how much data can be omitted, should surpass 0.50, but never fall below 0.25 (39). Provided that strength centrality boasts greater stability than either compactness and intermediation (43, 44), the current study adopted strength centrality as its primary index, which denotes the aggregate edge weights for every node, illustrating the potential activation interplay among symptoms (45). Network Comparison Test (NCT) in R-package was used to contrast symptomatic discrepancies between TRS and NTRS counterparts (46). The NCT leverages a permutation test to scrutinize the uniformity in overall strength (the aggregate of edge weights) and architecture across two distinct networks (47).

## 3 Results

### 3.1 Demographic and clinical characteristics between TRS and NTRS

Altogether, 511 schizophrenia patients were included in the analysis, divided into TRS and NTRS group. In the TRS (N = 269), 200 were males (69 females), with a mean age of 51.33 years (SD = 9.31), and in the NTRS (N = 242), 176 were males (66 females), with a mean age of 44.73 years (SD = 9.32). TRS group remained longer in the duration of the disease (M ± SD, 27.38 ± 11.16), and TRS group stayed longer duration of hospitalization(M ± SD, 5.58 ± 4.70). The mean total scores of scales were statistically different between TRS and NTRS, shown in **Table 1**.

**Table 1:** Comparison of demographic and clinical characteristics between TRS and NTRS.

Variables	TRS (N = 269)	NTRS (N = 242)	χ²/t	P
	N/M ± SD	N/M ± SD		
Gender (Male/Female)	200/69	176/66	0.17	0.689
Age	51.33 ± 9.31	44.73 ± 9.32	8.00	<0.001
Education Years	11.51 ± 2.78	12.05 ± 3.09	-2.08	0.039
Course of Disease	27.38 ± 11.16	20.11 ± 9.51	7.88	<0.001
Duration of Hospitalization	5.58 ± 4.70	2.78 ± 3.08	8.02	<0.001
Dosage (CPZ mg)	421 ± 174.78	376.45 ± 185.67	2.79	0.005
PANSS-P	15.85 ± 5.71	9.94 ± 3.30	14.12	<0.001
PANSS-N	20.02 ± 6.83	13.46 ± 20.02	12.96	<0.001
PANSS-G	32.28 ± 6.17	22.46 ± 3.66	21.59	<0.001
SAPS	14.39 ± 10.83	3.37 ± 4.37	14.78	<0.001
SANS	35.52 ± 18.03	19.05 ± 10.15	12.54	<0.001
SAS	1.81 ± 2.16	0.71 ± 1.52	6.6	<0.001
AIMS	1.23 ± 2.17	0.30 ± 0.776	6.27	<0.001
BARS	0.82 ± 1.43	0.50 ± 1.19	2.77	0.006
CDSS	1.36 ± 1.62	1.57 ± 1.72	-1.47	0.142
GAF	57.69 ± 12.15	68.00 ± 7.78	-11.28	<0.001

TRS, Treatment Resistant Schizophrenia; NTRS, Non-Treatment-Resistant Schizophrenia; CPZ, Chlorpromazine; PANSS-P, Positive and Negative Syndrome Scale-Positive; PANSS-N, Positive and Negative Syndrome Scale-Negative; PANSS-G, Positive and Negative Syndrome Scale- General Psychopathology; SAPS, Positive Symptom Assessment Scale; SANS, Scale for Assessment of Negative Symptoms; SAS, Simpson-Angus Scale; AIMS, Abnormal Involuntary Movements Scale; BARS, Barnes Akathisia Rating Scale; CDSS, Calgary Schizophrenia Depression Scale; GAF, Global Assessment of Functioning Scale.

### 3.2 Network structure and centrality measure analysis

**Figure 1:**
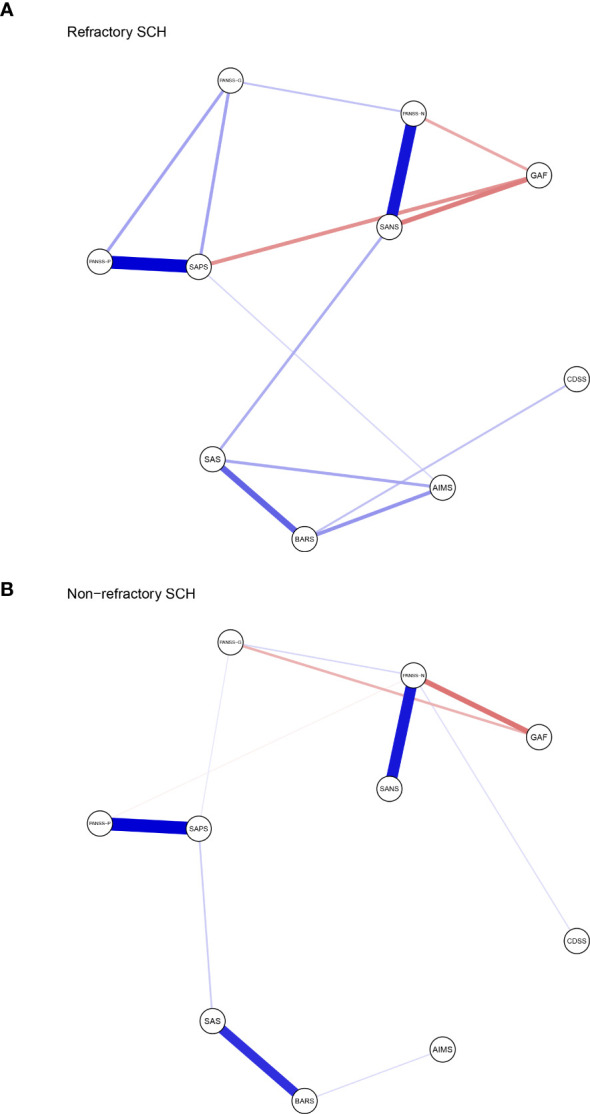
Network structure of TRS and NTRS. Network characteristics in **(A)** TRS group (N=269), **(B)** NTRS (N=242). Nodes represent respectively the score of PANSS-P, PANSS-N, PANSS-G, SAPS, SANS, SAS, AIMS, BARS, CDSS and GAF. PANSS-P, Positive and Negative Syndrome Scale-Positive; PANSS-N, Positive and Negative Syndrome Scale-Negative; PANSS-G, Positive and Negative Syndrome Scale- General Psychopathology; SAPS, Positive Symptom Assessment Scale; SANS, Scale for Assessment of Negative Symptoms; SAS, Simpson-Angus Scale; AIMS, Abnormal Involuntary Movements Scale; BARS, Barnes Akathisia Rating Scale; CDSS, Calgary Schizophrenia Depression Scale; GAF, Global Assessment of Functioning Scale. Edges represent partial correlations between symptoms. Edge width indicates the strength of the partial correlations. The red line represents a negative correlation, while the blue one represents a positive correlation.

**Figure 2:**
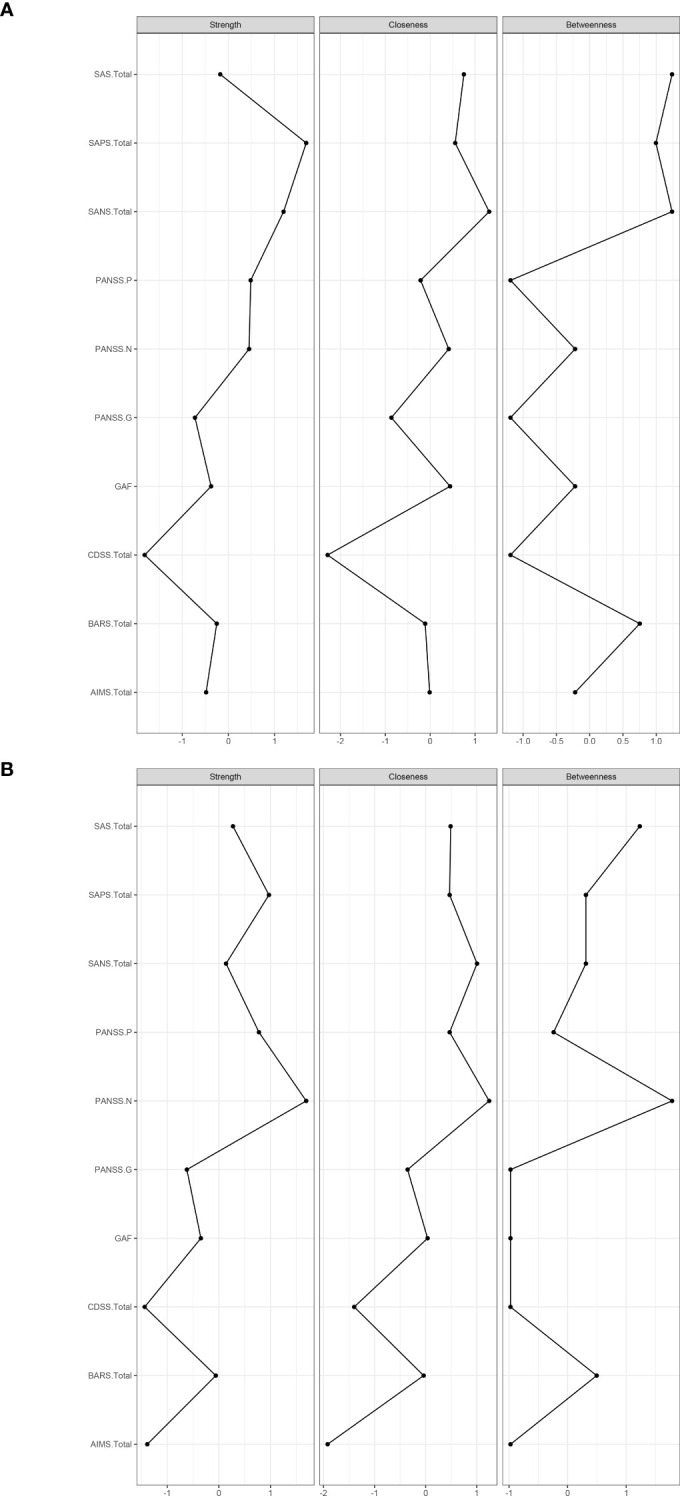
Centrality indices of symptoms (standardized z-scores). Centrality indices of symptoms of TRS **(A)**, and Centrality indices of symptoms of NTRS **(B)**.

The resulting network of 10 items representing schizophrenia symptoms (negative, positive, general psychopathology, depressive and function impairmen), and drug induced symptoms (extrapyramidal side effects and akathisia) was illustrated in **Figure 1**. The network in TRS (**Figure 1A**) and NTRS (**Figure 1B**) were well connected and had no isolated nodes (global strength: 4.798 and 3.402 respectively). The overall two architectures were significant difference (S = 1.396, *p* < 0.002) and nodes’ connection and edges’ strength were varied (M = 0.289, *p* < 0.009). The network of symptoms was organized around SAPS(connected to PANSS-P, PANSS-G and AIMS positively and to GAF negatively) in TRS group, which has the highest number of connections to other nodes. While in NTRS group, the symptoms were organized around PANSS-N connected to SANS, PANSS-G, CDSS positively and to GAF negatively (**Figure 1B**). In the network comparison, there were significant difference on 9 edges, SAPS to GAF (*p <*0.000), SANS to GAF (*p <*0.000), PANSS-P to PANSS-N (*p* =0.002), PANSS-P to AIMS (*p* =0.003), SAPS to SANS (*p* =0.008), PANSS-P to PANSS-G (*p* =0.02), PANSS-P to GAF (*p* =0.041) between TRS and NTRS (**Supplementary Table 1**) and 5 nodes, SAPS (*p <*0.000), SANS (*p <*0.000), AIMS (*p <*0.000), GAF (*p* =0.002) and PANSS-G (*p* =0.006), all *p <*0.01, (**Supplementary Table 2**). The highest closeness and betweenness index in TRS group is SANS (**Figure 2A**), while for strength is SAPS (**Figure 2A**). PANSS-N has highest index of all three centrality dgreees (**Figure 2B**) in NTRS group.

### 3.3 Network accuracy and stability

**Supplementary Figure 1** displayed the resulting plots and revealed substantial bootstrapped confidence intervals (CIs) around the estimated edge weights. This suggested that many edge weights likely do not significantly differ from one another. The slimmer bootstrapped CIs indicated that the order of most edges in the network should be interpreted well. The CS coefficient (**Supplementary Figure 2**) demonstrated the high stability of strength centrality measure with CS value of 0.75 in TRS group, and 0.752 in NTRS group.

## 4 Discussion

TRS is associated with multiple clinical features, including poor premorbid social functioning, longer duration of untreated psychosis (48–50), baseline PANSS score (51), heritability (52) and cognitive profile (53). Multiple clinical features differences between TRS and NTRS were identified. For TRS, it is essential to address both negative and positive symptoms in our interventions, particularly focusing on the impact of negative symptoms on the patient’s functioning.

The findings in current study indicated that multiple demographic and clinical features differ between TRS and NTRS, except gender and CDSS. In line with the results of others which found male sex is not associated with TRS (17, 54, 55). A cohort study demonstrated that men were one and a half times as likely as women to develop TRS (56). Absence of depression may indicate worse functioning (57). Although absence Statistical difference (*p* = 0.142) of depression (as indicated by CDSS score) between TRS and NTRS in our study, an increase in CDSS scores was also seen in our NTRS group. As shown in **Table 1**, TRS patients often require higher doses and more complex medication regimens, including the use of clozapine, which is considered a treatment of last resort for those who do not respond to other antipsychotics (58–60). This complexity of TRS and the severe burden it imposes on society and families encourages us to further explore the core symptoms of which and how they interact with each other.

In TRS group, SAPS connects frequently to other symptoms, which positively correlated with PANSS-P, PANSS-G and AIMS with a decline strength (**Figure 1A**). In line with these findings, TRS showed the highest loadings in the positive symptoms (2, 61). The analysis of the TRS group reveals intricate relationships between various symptom assessment scales. SAPS shows notable correlations with other scales, offering insights into symptomatology and treatment outcomes. The highest strength centrality SAPS indicated which might be as central to the network (38). The SAPS positively correlates with PANSS-G, indicating that patients with higher SPAS scores also exhibit significant general psychiatric symptoms (62). It also shows a positive correlation with the AIMS, albeit with declining strength, suggesting a connection between positive symptoms and motor abnormalities, possibly reflecting the side effects of high dose antipsychotic treatments. Conversely, SAPS negatively correlates with the GAF, indicating that higher positive symptoms are associated with lower overall functioning (63). This highlights the need for treatment strategies that address both symptom severity and functional impairment to improve patients’ quality of life. This complex interplay highlights the challenges in managing TRS, where treatments aimed at reducing positive symptoms can influence motor functions differently. The highest loadings in positive symptoms among TRS patients underscore the persistence of these symptoms despite treatment, emphasizing the need for comprehensive assessment tools and tailored treatment strategies that address both positive symptoms and general psychopathology while balancing medication efficacy and side effects. With the highest closeness and betweenness index of SANS in TRS, which suggested that this symptoms can affect changes in other parts of the network quickly (64), and it is important in the connection that the other symptoms have between SANS (65, 66). These results suggest that effective control negative symptoms of TRS may rapidly improve other symptoms.

While in NTRS group, GAF and PANSS-P exhibit a negative correlation with PANSS-N, indicating that as negative symptoms increase, overall functioning decreases and positive symptoms might decrease as well (67). This suggests that severe negative symptoms significantly impair daily activities, social interactions, and occupational performance, while also possibly reflecting different underlying mechanisms or treatment effects influencing symptom domains (68, 69). The complex interplay of these symptoms highlights the need for comprehensive treatment approaches that address not only negative symptoms but also general psychopathology, depression, and positive symptoms. Enhancing social and occupational functioning should be a key component of treatment plans to improve the quality of life for NTRS patients (70). The node PANSS-N has highest index of all three centrality dgreees (**Figure 2B**), partly differ from TRS network, which reflects the complex differences between the two types of schizophrenia. Further research should investigate these relationships to develop more effective management strategies of negative symptoms in NTRS.

McMahon and colleagues (71) proposing that factors: reality distortion (grandiosity, suspiciousness, hallucinatory behavior and unusual thought content), disorganization (conceptual disorganization, mannerism and posturing, disorientation), negative symptoms (emotional withdrawal, motor retardation and blunted affect), and anxiety/depression (anxiety, guilty feelings and depression) should be used for the analysis of data of clinical trials involving patients with TRS. Four dimensions: negative/disorganization (emotional withdrawal, disorientation, blunted affect, mannerisms and posturing, conceptual disorganization), excitement (excitement, hostility, tension, grandiosity and uncooperativeness), positive (unusual thought content, suspiciousness and hallucinatory behavior), and depression (depression, guilt feelings and motor retardation) were found importment in TRS by anather colleague (72). Mutiple studies focused on the bioligcal differences between TRS and NTRS (73–78). However, few studies have explored the differences in clinical features’ networks between the two groups, which are more instructive for clinical treatment. The current network analysis compared schizophrenia symptoms and drug-induced symptoms between TRS and NTRS groups. The networks in TRS and NTRS showed significant differences in structure, with TRS exhibiting stronger overall connectivity and organization around positive symptoms (assessed by SAPS), as well as the negative symptoms(assessed by SANS). This suggests that more attention should be paid to positive symptoms and negative symptoms in the clinical treatment of TRS. Significant differences were also found in specific edges and nodes between the groups, suggesting distinct patterns of symptom interaction and severity. These differences in TRS and NTRS groups highlight distinct patterns of symptom interactions and severity that may contribute to treatment resistance. Specifically, differences in edges like PANSS.P - PANSS.N and PANSS.P - PANSS.G suggest unique relationships between positive, negative, and general psychopathology symptoms in TRS versus NTRS (79–81). Additionally, variations in nodes such as PANSS.G, SAPS, SANS, AIMS, and GAF underscore differences in symptom domains and functional outcomes between the groups. Due to the differences in treatment options between TRS and NTRS, their side effects can vary depending on the choice of drug and dose (48, 82, 83). In summary, current study found the core symptoms and network of symptoms-side effects interaction differ between TRS and NTRS.

This study has several limitations that should be acknowledged. First, the sampling method employed, while convenient, may introduce sampling bias. Second, a single hospital in Beijing and small sample size and the fact that all participants may limit the generalizability of the findings to the wider population of interest. Third, some of the scales we have used are outdated, and some new scales have begun to be use, such as the Brief Negative Symptom Scale (BNSS) and Clinical Assessment Interview for Negative Symptoms (CAINS). Finally, as the researchers are medical staff, the survey design may have been influenced by clinical observation bias. Future research could benefit from integrating clinical observation with established theoretical frameworks to address these limitations.

## 5 Conclusion

Treatment resistance in schizophrenia is multifaceted. There is no single definition that encompasses all aspects, as the pathogenesis is not well understood and the disease remains incompletely characterized. Positive and negative symptoms are core clinical features of TRS. The differences in core symptoms between TRS and NTRS may partly explain the resistance to treatment in TRS. Managing both positive and negative symptoms in TRS remains a crucial task, with particular attention needed for negative symptoms and related clinical features.

## Data Availability

The original contributions presented in the study are included in the article/**Supplementary Material**. Further inquiries can be directed to the corresponding authors.
