# Supplementary material for: Network analysis of clinical features in patients with treatment-resistant schizophrenia
Source: Front Psychiatry. 2025 Feb 6;16:1537418. doi: 10.3389/fpsyt.2025.1537418 (PMC11839625; doi:10.3389/fpsyt.2025.1537418)
Supplement: Supplementary file 1 [file DataSheet1.pdf]

supplementary materials

Table S1. The difference in edges between TRS and NTRS

Node 1	Node 2	P
SAPS	GAF	0.000
SANS	GAF	0.000
PANSS.P	PANSS.N	0.002
PANSS.P	AIMS	0.003
SAPS	SANS	0.008
PANSS.P	PANSS.G	0.020
PANSS.P	GAF	0.021
SAPS	AIMS	0.037
PANSS.G	GAF	0.041
PANSS.P	SANS	0.074
SAS	AIMS	0.105
GAF	CDSS	0.119
GAF	CDSS	0.119
PANSS.N	BARS	0.121
SANS	SAS	0.124
PANSS.N	GAF	0.127
PANSS.P	BARS	0.142
PANSS.G	SAPS	0.152
BARS	CDSS	0.183
BARS	CDSS	0.183
PANSS.P	CDSS	0.190
PANSS.G	AIMS	0.200
PANSS.G	CDSS	0.205
SAS	BARS	0.228
PANSS.N	CDSS	0.246
AIMS	BARS	0.276
SAS	GAF	0.303
SAPS	SAS	0.319
PANSS.N	SANS	0.396
SANS	AIMS	0.455
SAPS	BARS	0.493
SAS	CDSS	0.507
PANSS.P	SAPS	0.517
PANSS.N	PANSS.G	0.588
SANS	BARS	0.663
SAPS	CDSS	0.709
PANSS.G	SANS	0.776
AIMS	GAF	0.843
AIMS	CDSS	0.890

PANSS.N	SAPS	1.000
PANSS.P	SAS	1.000
PANSS.N	SAS	1.000
PANSS.G	SAS	1.000
PANSS.N	AIMS	1.000
PANSS.G	BARS	1.000
BARS	GAF	1.000
SANS	CDSS	1.000

PANSS-P: Positive and Negative Syndrome Scale-Positive

PANSS-N: Positive and Negative Syndrome Scale-Negative

PANSS-G: Positive and Negative Syndrome Scale- General Psychopathology.

SAPS: The Positive Symptom Assessment Scale

SANS: The Scale for Assessment of Negative Symptoms

SAS: The Simpson-Angus Scale

AIMS: The Abnormal Involuntary Movements Scale

BARS: The Barnes Akathisia Rating Scale

CDSS: The Calgary Schizophrenia Depression Scale

GAF: The Global Assessment of Functioning Scale

Table S2. The difference in nodes between TRS and NTRS

Node	P
SAPS	0.000
SANS	0.000
AIMS	0.000
GAF	0.002
PANSS.G	0.006
PANSS.P	0.270
SAS	0.301
BARS	0.320
CDSS	0.327
PANSS.N	0.451

PANSS-P: Positive and Negative Syndrome Scale-Positive

PANSS-N: Positive and Negative Syndrome Scale-Negative

PANSS-G: Positive and Negative Syndrome Scale- General Psychopathology.

SAPS: The Positive Symptom Assessment Scale

SANS: The Scale for Assessment of Negative Symptoms

SAS: The Simpson-Angus Scale

AIMS: The Abnormal Involuntary Movements Scale

BARS: The Barnes Akathisia Rating Scale

CDSS: The Calgary Schizophrenia Depression Scale

GAF: The Global Assessment of Functioning Scale

a

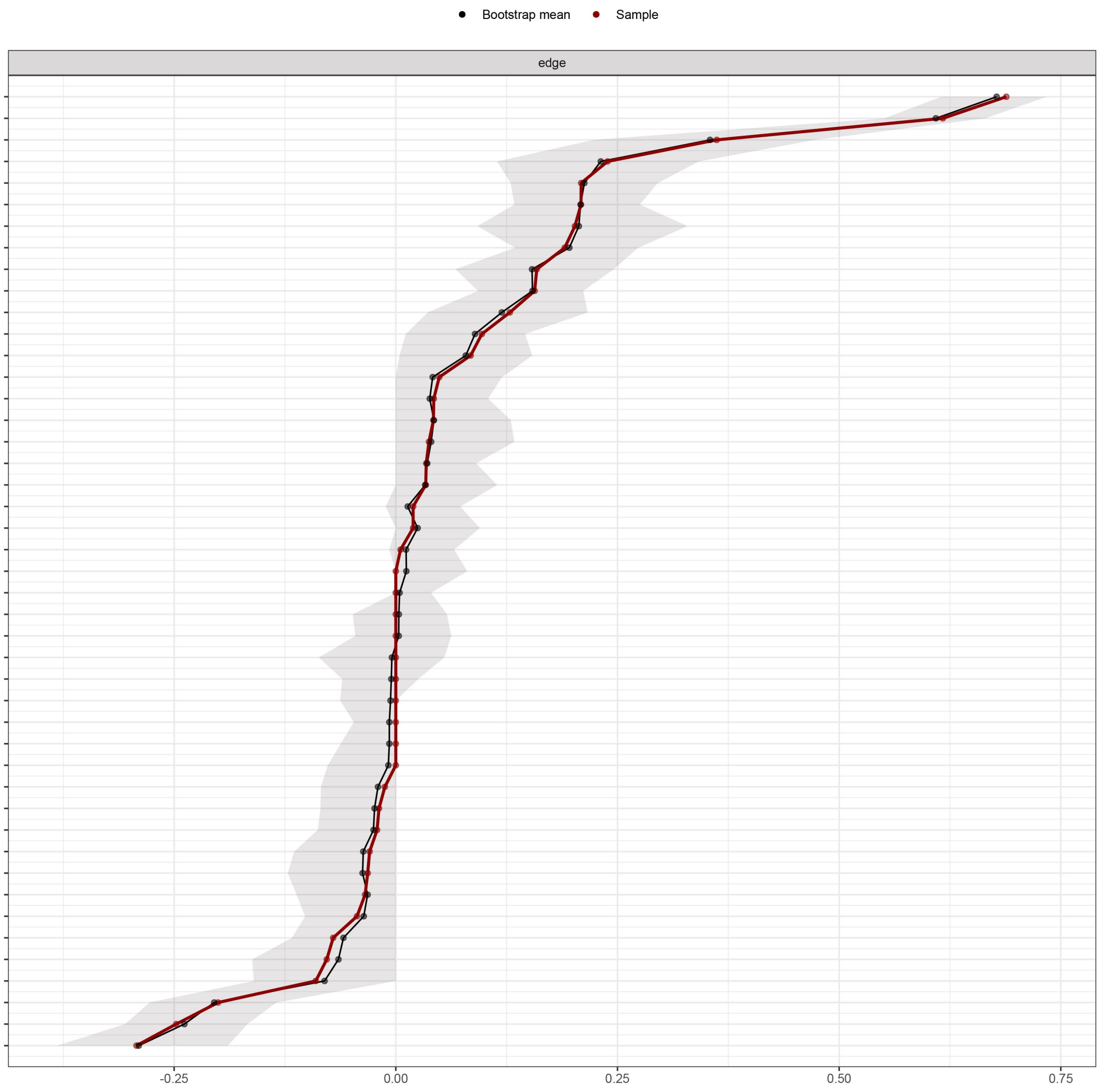

b

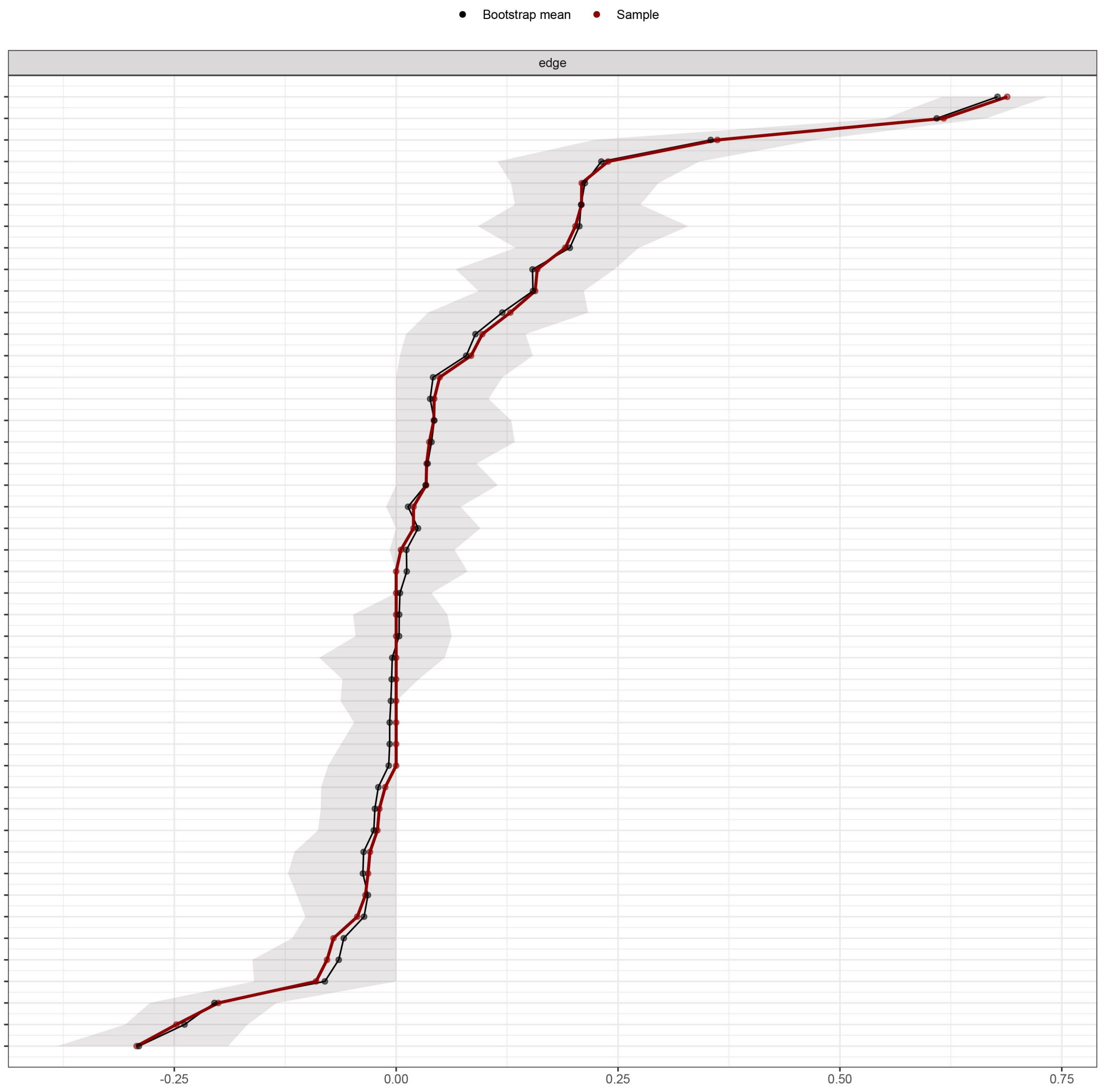

Supplementary Figure 1 **Bootstrapped confidence intervals of estimated edge-weights.** TRS group (a), NTRS group (b). The red line indicates the sample values and the gray area the bootstrapped CIs.

a

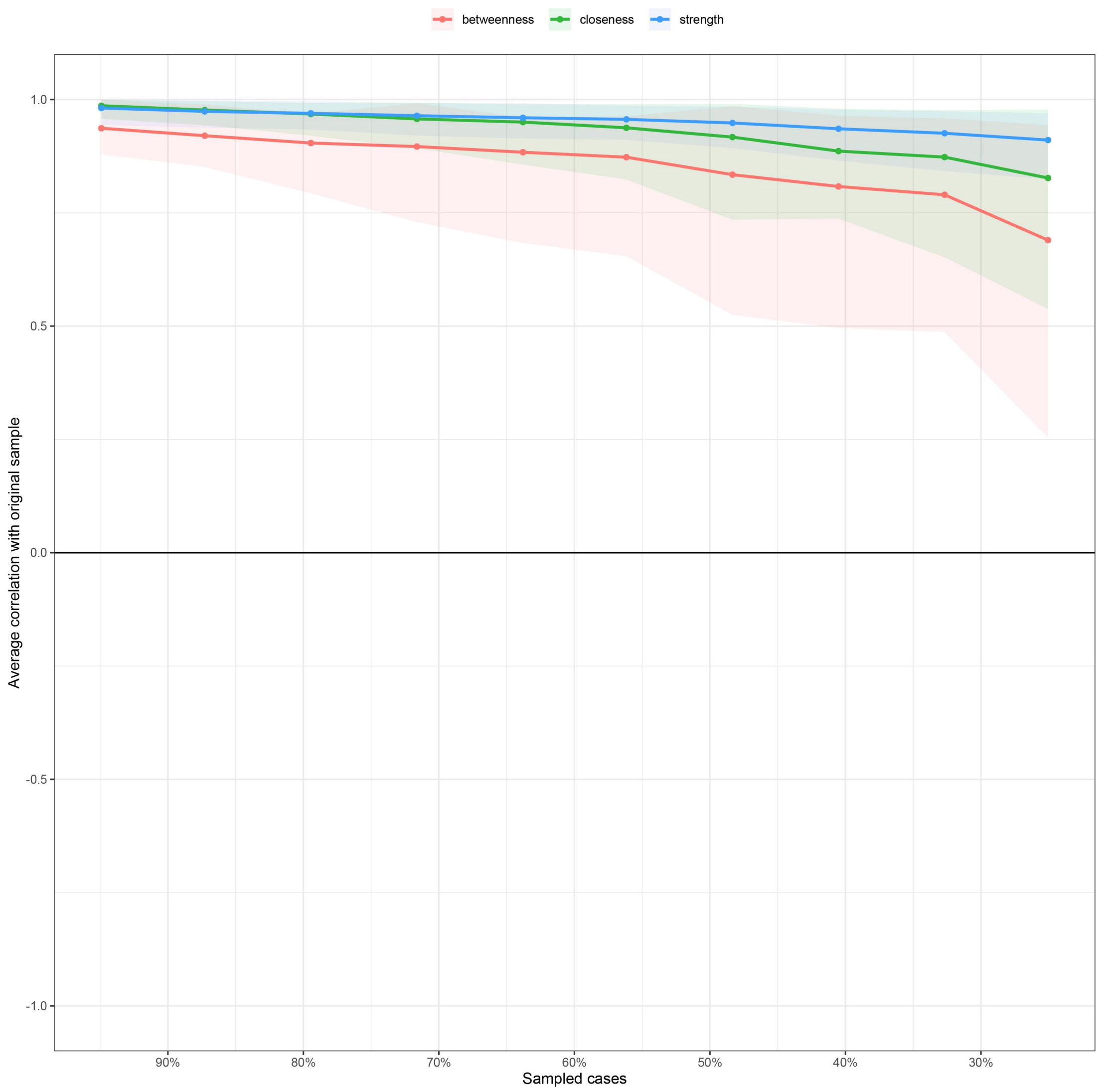
betweenness
closeness
strength
1.0
0.5
0.0
-0.5
-1.0
Average correlation with original sample
90%
80%
70%
60%
50%
40%
30%
Sampled cases

b

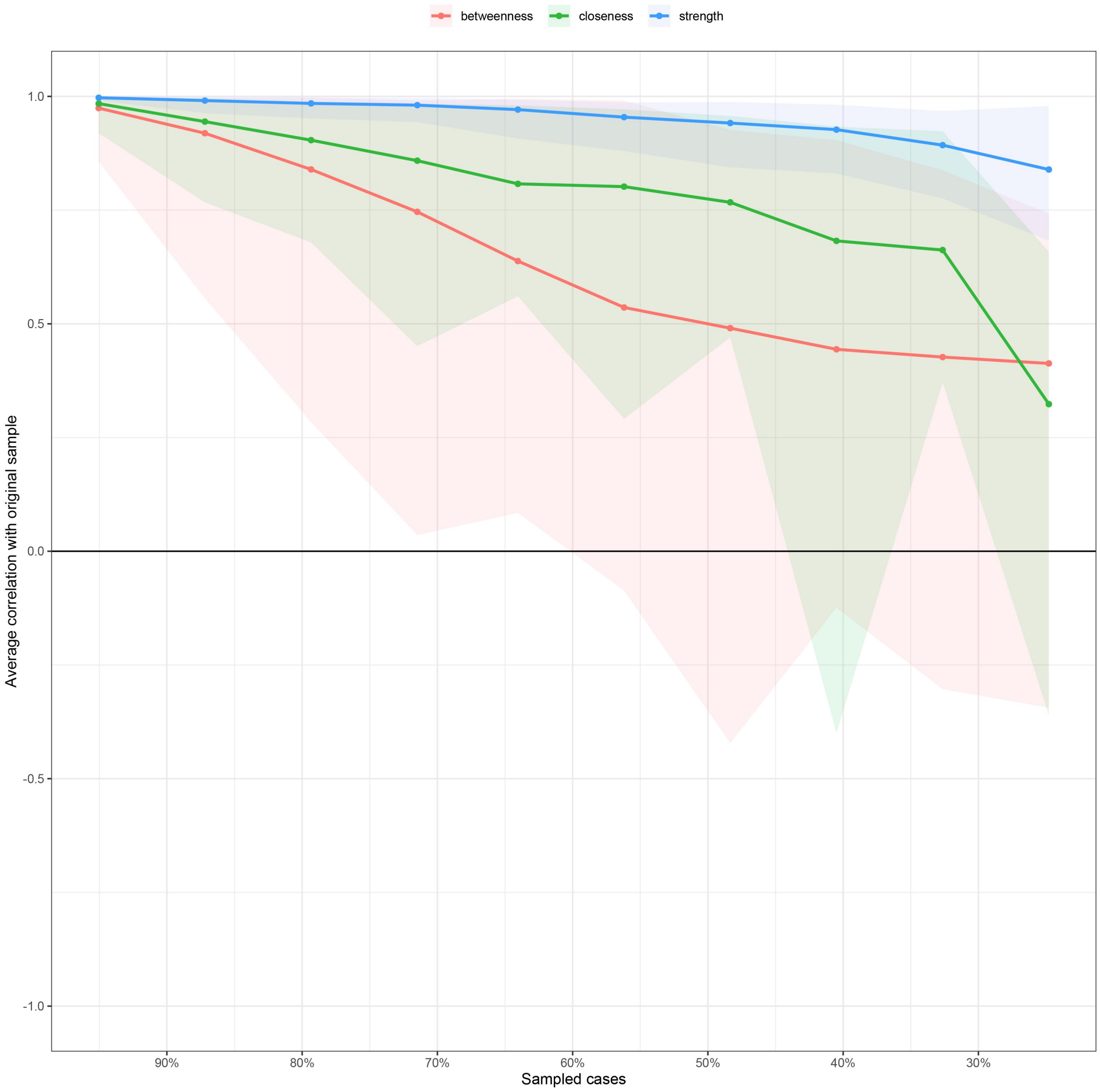

Supplementary Figure 2 **Stability of centrality indices.** Stability of centrality indices by case dropping subset bootstrap of TRS group (a). Stability of centrality indices by case dropping subset bootstrap of NTRS group (b). Each line indicates the correlations of betweenness, closeness and strength, while areas indicate 95% CI.
